# Supplementary material for: Multiplex design and discovery of proximity handles for programmable proteome editing
Source: bioRxiv. 2025 Oct 13:2025.10.13.681693. Preprint. [Version 1] doi: 10.1101/2025.10.13.681693 (PMC12632987; doi:10.1101/2025.10.13.681693)
Supplement: 8 [file NIHPP2025.10.13.681693v1-supplement-8.pdf]

bioRxiv preprint doi: https://doi.org/10.1101/2025.10.13.681693; this version posted October 13, 2025. The copyright holder for this preprint (which was not certified by peer review) is the author/funder, who has granted bioRxiv a license to display the preprint in perpetuity. It is made available under aCC-BY 4.0 International license.

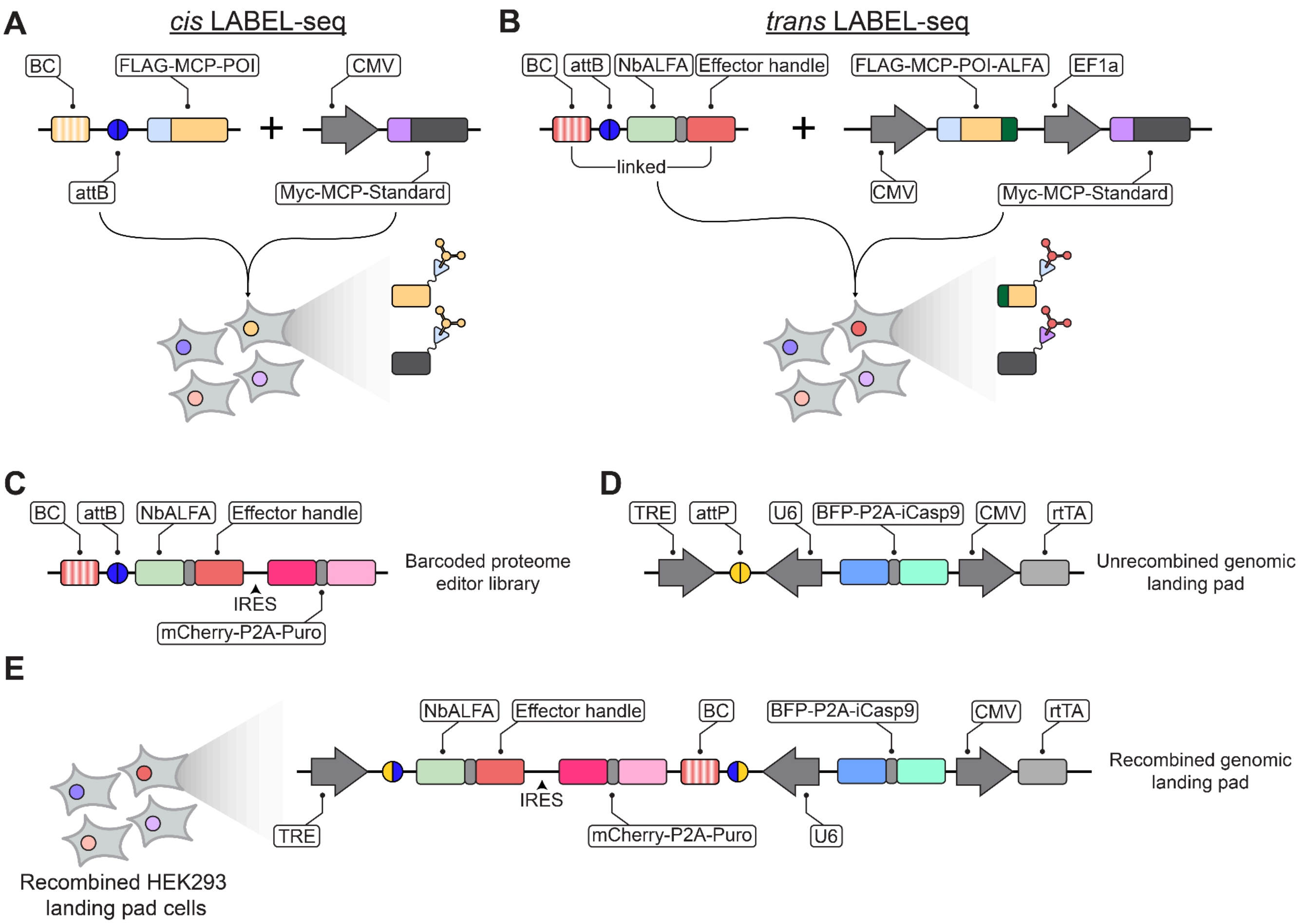


**Figure S1. Schematic of genomic landing pad and *cis* versus *trans* LABEL-seq assays**

**A)** Diagram of *cis* LABEL-seq in which the barcode (BC) and FLAG-MCP-POI protein are encoded from the same DNA molecules. **B)** Diagram of *trans* LABEL-seq in which barcode (BC) and FLAG-MCP-POI-ALFA protein are encoded from distinct DNA molecules. **C)** A barcoded proteome editor library prior to recombination at the landing pad. The barcoded proteome editor library also has a mCherry-P2A-Puro cassette that is translated from an upstream internal ribosome entry site (IRES). **D)** Diagram showing the single-copy genomic landing pad prior to recombination, as well as a barcoded element library. The landing pad contains an attP site flanked by convergent tetracycline response element (TRE) and U6 promoters, a BFP-P2A-iCasp9 cassette, and a CMV-driven reverse tetracycline transactivator (rtTA). A barcoded element library with an attB site can be recombined with the genomic attP site via Bxb1-mediated recombination. **E)** Diagram showing the single-copy genomic landing pad following recombination, which places the library element and IRES-mCherry-P2A-Puro cassette under control of the TRE promoter and the circular RNA barcode under control of the U6 promoter.

bioRxiv preprint doi: https://doi.org/10.1101/2025.10.13.681693; this version posted October 13, 2025. The copyright holder for this preprint (which was not certified by peer review) is the author/funder, who has granted bioRxiv a license to display the preprint in perpetuity. It is made available under aCC-BY 4.0 International license.

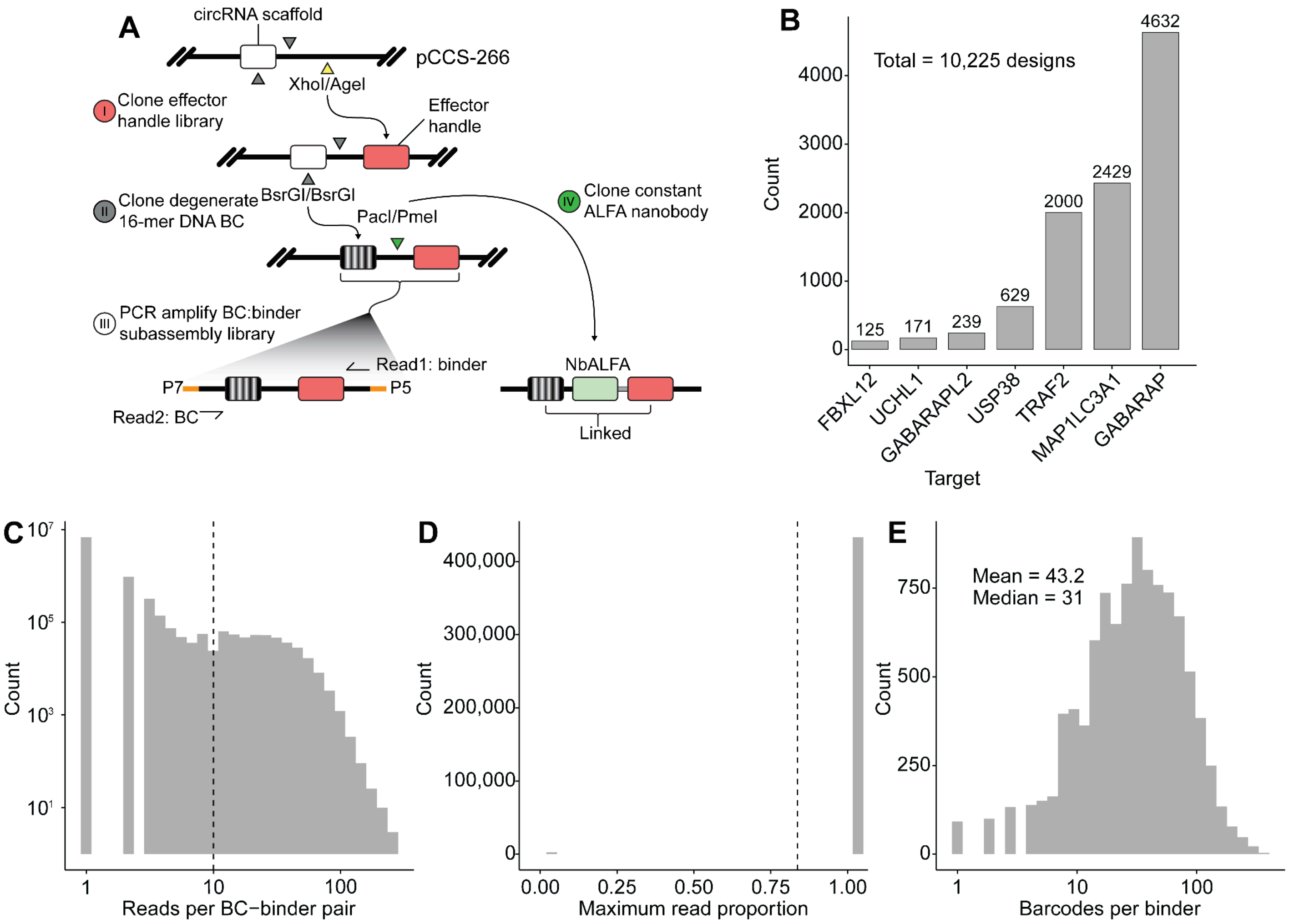


**Figure S2. Plasmid library composition and characterization**

**A)** Schematic detailing how the barcoded binder library was cloned, including subassembly. **B)** Barplot showing the number of designs generated for each target effector. **C)** Histogram showing the distribution of the number of sequencing reads per barcode-binder pair. The vertical dashed line indicates the threshold above which barcode-binder pairs were considered valid. **D)** Histogram showing the maximum read proportion of individual barcodes. For an individual barcode, the proportion of reads associated with any binder was computed to ascertain the fidelity of barcode binder association. The vast majority of barcodes were associated with a single binder. **E)** Histogram showing the distribution of the number of barcodes associated with each binder.

bioRxiv preprint doi: https://doi.org/10.1101/2025.10.13.681693; this version posted October 13, 2025. The copyright holder for this preprint (which was not certified by peer review) is the author/funder, who has granted bioRxiv a license to display the preprint in perpetuity. It is made available under aCC-BY 4.0 International license.

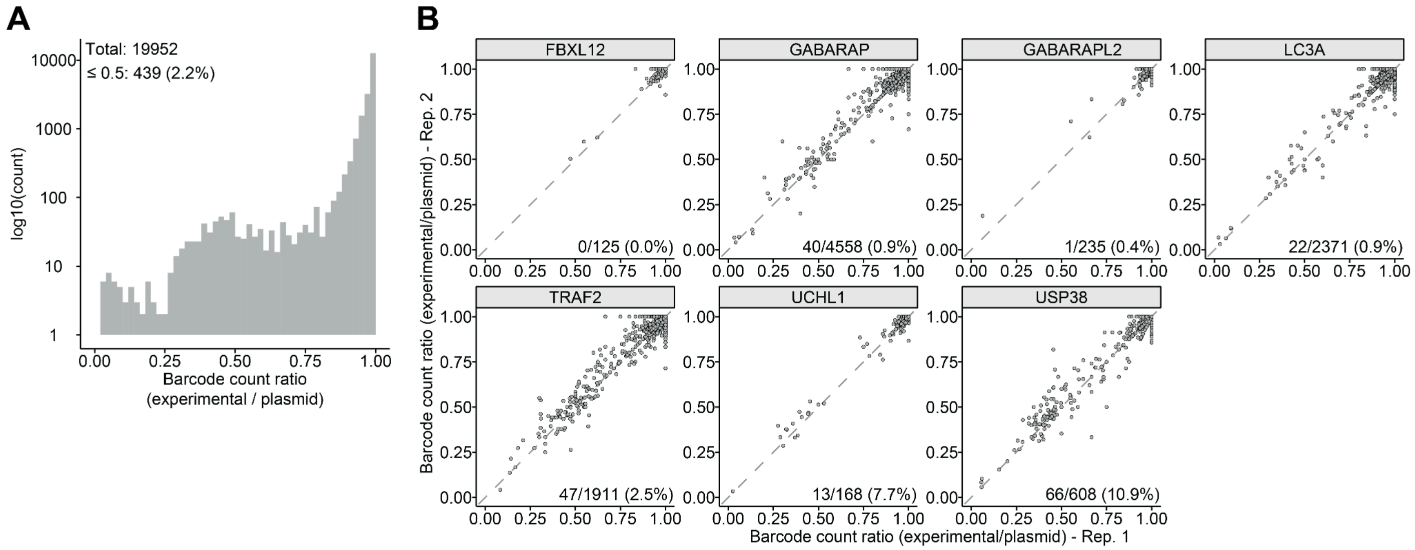


**Figure S3. Comparison of plasmid and experimental barcodes**

**A)** Histogram showing distribution of the ratio of experimentally recovered barcodes to plasmid library barcodes for each binder across two transfection replicates. The total number of binders represented in the plot is indicated, as well as the number and percentage of binders with a ratio ≤ 0.5 in either of the two replicates. The distribution suggests that some binders are “dropping out” of the experiment when expressed in cells. **B)** Scatter plots comparing the ratio of experimentally recovered barcodes to plasmid library barcodes for each binder between the two transfection replicates. Each point represents an individual binder. For each effector target, the number of binders with a ratio ≤ 0.5 in both replicates is indicated, as well as the percentage. The patterns suggest that the drop-out phenomenon is reproducible between replicates, and moreover is more likely to occur with binders designed against some effectors (*e.g.* USP38; 10.9%) than others (*e.g.* FBXL12; 0%).

bioRxiv preprint doi: https://doi.org/10.1101/2025.10.13.681693; this version posted October 13, 2025. The copyright holder for this preprint (which was not certified by peer review) is the author/funder, who has granted bioRxiv a license to display the preprint in perpetuity. It is made available under aCC-BY 4.0 International license.

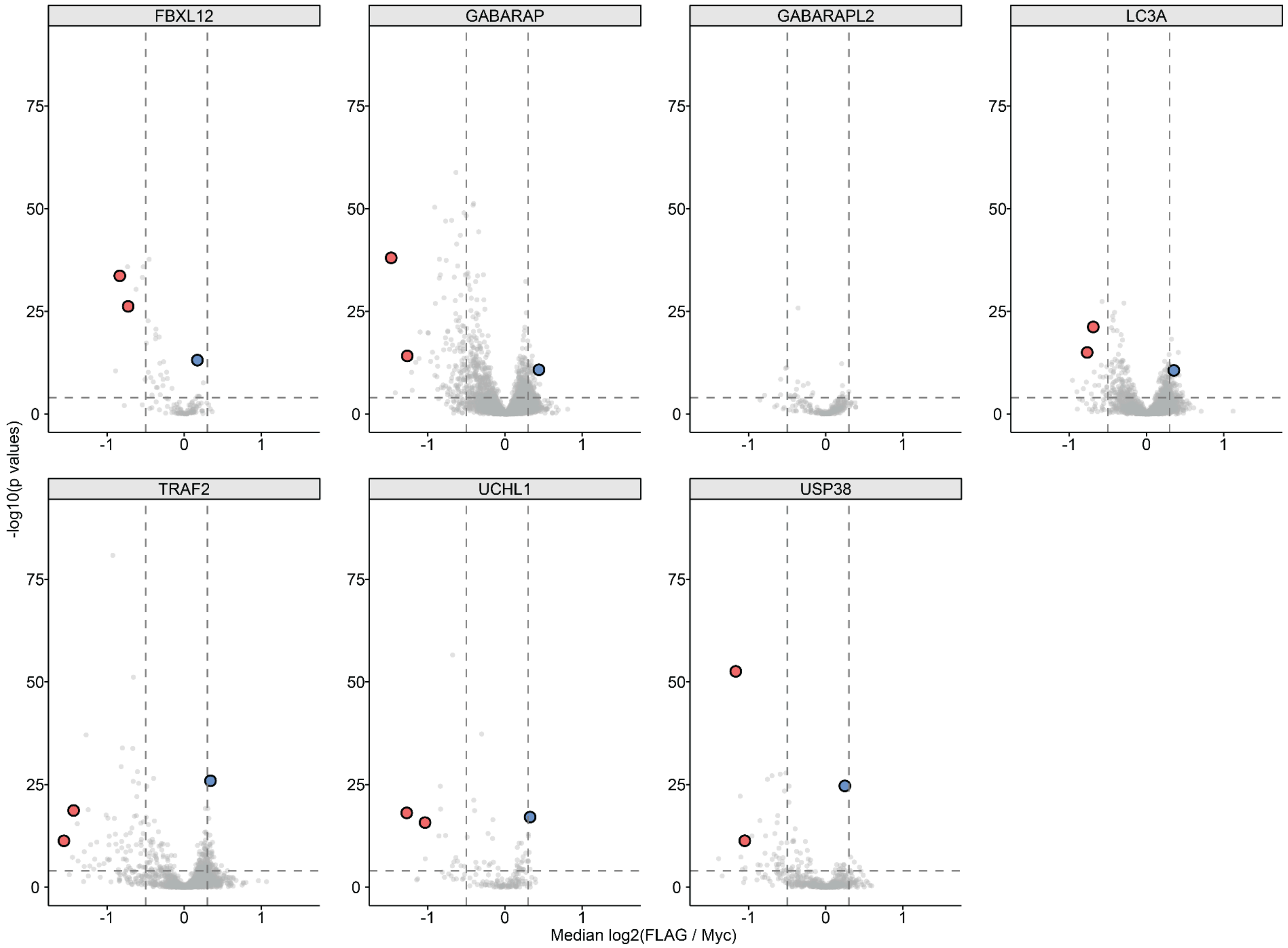


**Figure S4. Assessment of hit rate per effector and selection of effector handles for validation experiments.**

Volcano plots comparing the distribution of effect sizes (x-axes) and significance (y-axes) faceted by effector. Degrader (red points) and stabilizer (blue points) effector handles selected for validation are indicated.

bioRxiv preprint doi: https://doi.org/10.1101/2025.10.13.681693; this version posted October 13, 2025. The copyright holder for this preprint (which was not certified by peer review) is the author/funder, who has granted bioRxiv a license to display the preprint in perpetuity. It is made available under aCC-BY 4.0 International license.

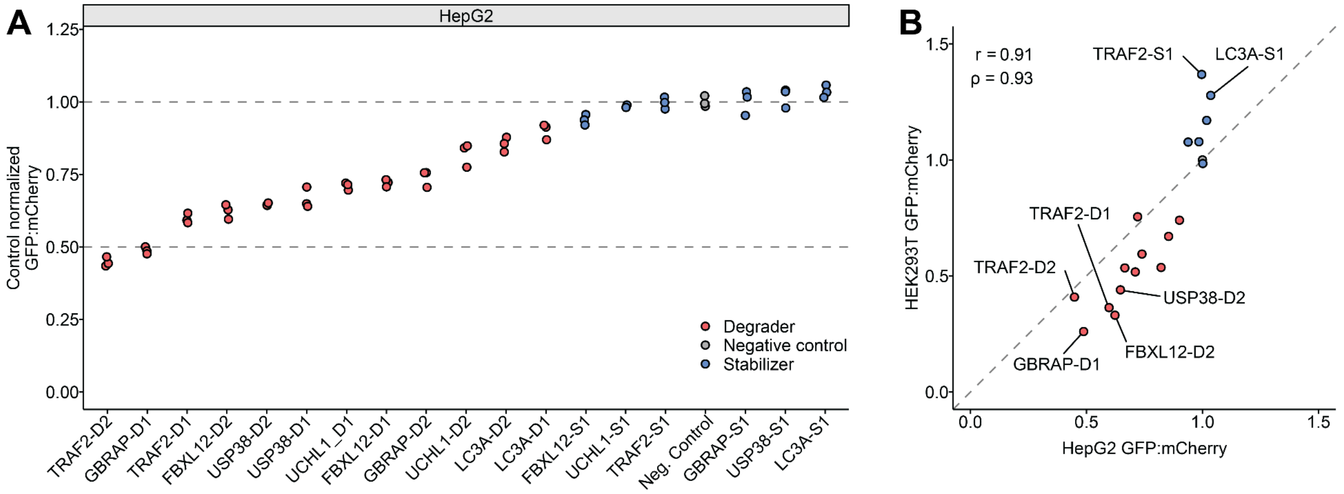


**Figure S5. Binder-induced GCK degradation validates in an alternative cell line.**

**A)** Dot plot of control-normalized GFP:mCherry ratios measured in HepG2 cells expressing a GFP-GCK-ALFA reporter and indicated candidate effector designs. Each point represents a transfection replicate and the dashed line at 1 indicates the mean of the negative-control effector (grey points). Effectors are ordered along the x-axis by mean normalized GFP:mCherry ratio. **B)** Scatterplot comparing measured effects from the singleton validation in HepG2 cells (x-axis) vs. HEK293T cells (y-axis). Pearson (r) and Spearman (ρ) correlation values are shown.

bioRxiv preprint doi: https://doi.org/10.1101/2025.10.13.681693; this version posted October 13, 2025. The copyright holder for this preprint (which was not certified by peer review) is the author/funder, who has granted bioRxiv a license to display the preprint in perpetuity. It is made available under aCC-BY 4.0 International license.

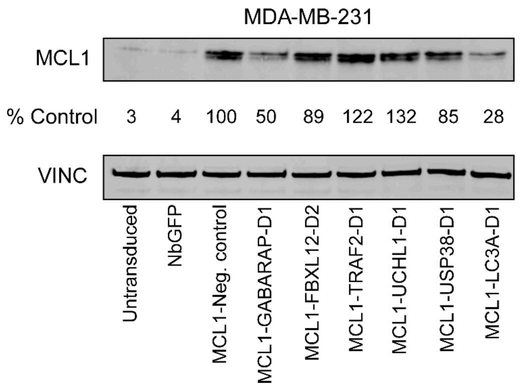


**Figure S6. Assessment of effects of bifunctional MCL1-effector binder fusions on endogenous MCL1 levels.**

Western blot analysis of MCL1 levels in MDA-MB-231 cells, 72 hrs after transduction with indicated MCL1-effector binder fusions. Untransduced and GFP-nanobody (NbGFP) transduced cells serve as negative controls.

bioRxiv preprint doi: https://doi.org/10.1101/2025.10.13.681693; this version posted October 13, 2025. The copyright holder for this preprint (which was not certified by peer review) is the author/funder, who has granted bioRxiv a license to display the preprint in perpetuity. It is made available under aCC-BY 4.0 International license.

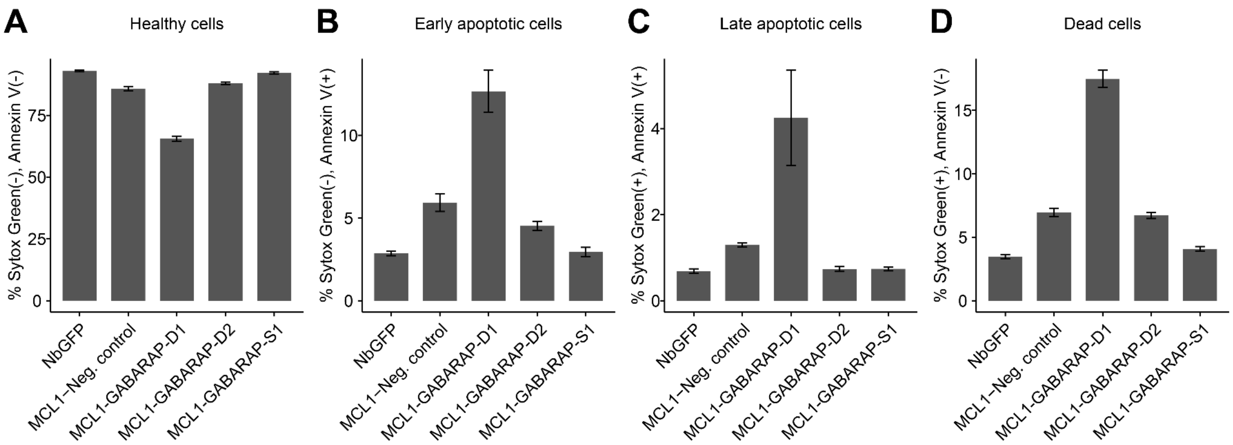


**Figure S7. Characterization of apoptosis induction by bifunctional MCL1-GABARAP binder fusions**

**A-D**) Bar plots showing the percentage (y-axis) of cells in four states in the presence of various MCL1-effector binder fusions (x-axis). States were defined as: healthy (**A**), early apoptotic (**B**), late apoptotic (**C**), and dead (**D**) based on the indicated flow cytometry parameters (y-axis labels). Bars indicate the mean of three transduction replicates, error bars indicate ± SEM.
